# Biological Activities and Potential Oral Applications of N-Acetylcysteine: Progress and Prospects

**DOI:** 10.1155/2018/2835787

**Published:** 2018-04-22

**Authors:** Yanping Pei, Huan Liu, Yi Yang, Yanwei Yang, Yang Jiao, Franklin R. Tay, Jihua Chen

**Affiliations:** ^1^Stomatology Department of Navy General Hospital, Beijing 100048, China; ^2^State Key Laboratory of Military Stomatology & National Clinical Research Center for Oral Diseases & Shaanxi Key Laboratory of Oral Diseases, Department of Prosthodontics, School of Stomatology, The Fourth Military Medical University, Xi'an 710032, China; ^3^Department of Neurosurgery, PLA Army General Hospital, Beijing 100700, China; ^4^Department of Stomatology, Lanzhou General Hospital, Lanzhou Military Area Command of Chinese PLA, Lanzhou, Gansu 730050, China; ^5^Department of Stomatology, PLA Army General Hospital, Beijing 100700, China; ^6^Department of Endodontics, The Dental College of Georgia, Augusta University, Augusta, GA 30912, USA

## Abstract

N-Acetylcysteine (NAC), a cysteine prodrug and glutathione (GSH) precursor, has been used for several decades in clinical therapeutic practices as a mucolytic agent and for the treatment of disorders associated with GSH deficiency. Other therapeutic activities of NAC include inhibition of inflammation/NF-*κ*B signaling and expression of proinflammatory cytokines. N-Acetylcysteine is also a nonantibiotic compound possessing antimicrobial property and exerts anticarcinogenic and antimutagenic effects against certain types of cancer. Recently, studies describing potentially important biological and pharmacological activities of NAC have stimulated interests in using NAC-based therapeutics for oral health care. The present review focused on the biological activities of NAC and its potential oral applications. The potential side effects of NAC and formulations for drug delivery were also discussed, with the intent of advancing NAC-associated treatment modalities in oral medicine.

## 1. Introduction

**Figure 1:**
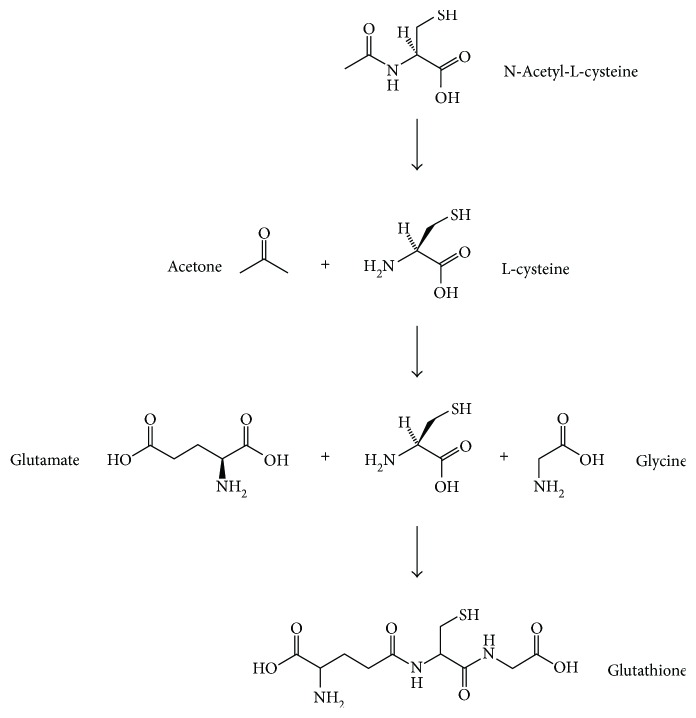
Chemical formula of N-acetyl cysteine and its conversion to glutathione.

N-acetylcysteine (NAC) possesses therapeutic effects over a wide range of disorders. These disorders include cystic fibrosis, acetaminophen poisoning, chronic obstructive pulmonary disease, chronic bronchitis, doxorubicin-induced cardiotoxicity, human immunodeficiency virus infection, heavy metal toxicity, and psychiatric/neurological disorders [1]. Being a N-acetyl derivative of the amino acid L-cysteine, NAC is a cysteine prodrug and glutathione (GSH) precursor that helps scavenge free radicals and bind metal ions into complexes [1] (Figure 1). Because NAC possesses anti-inflammatory activity via inhibition of nuclear factor kappa-light-chain-enhancer of activated B cells (NF-*κ*B) and modulation of proinflammatory cytokine synthesis [2], it has been used for modulating oxidative stress- and inflammation-related diseases [3]. Although NAC is not an antibiotic, it possesses antimicrobial properties and breaks down bacterial biofilms of medically relevant pathogens [4]. These characteristics render NAC a potential candidate for managing oral diseases.

**Figure 2:**
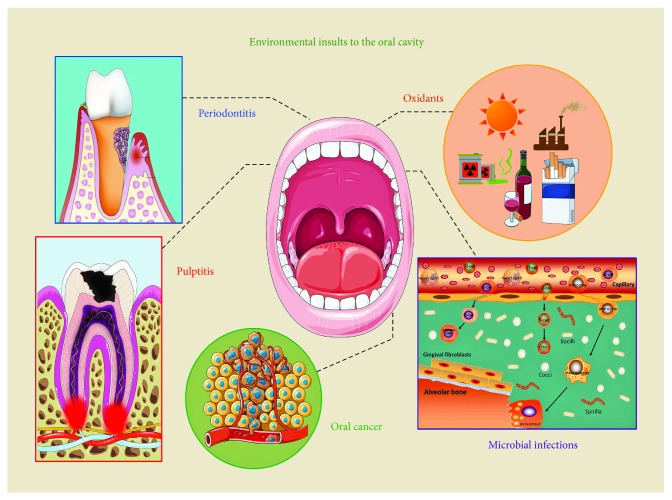
The oral cavity is exposed to different forms of environmental insults, including toxic chemicals, microbial infections, and mechanical injury. These insults generate oxidative stress, induce inflammation, and may even instigate cancer.

The oral cavity is the first point of entry for different forms of environmental insults, including toxic chemicals, microbial infections, and mechanical injury. These insults generate oxidative stress, induce inflammation, and may even initiate cancer (Figure 2). Some dental materials such as resins, metals, and ceramics are cytotoxic and have the potential to induce oxidative stress, DNA damage, inflammatory reactions, and cell death via apoptosis [5–7]. Disturbances in the regulation of the host inflammatory responses to bacterial infection in the dental pulp and periodontal tissues result in pulpitis and periodontitis [8]. Cigarette smoking, alcohol consumption, and betel nut chewing increase the risk of oral cancer [9]. Mechanical stresses produced during physiological masticatory activities, orthodontic tooth movement, or occlusal trauma, as well as heat stresses caused by tooth cavity preparation, light-initiated resin polymerization, or laser irradiation, may create oxidative stresses and inflammatory reactions in the dental pulp, resulting in pulpal necrosis [10, 11]. Hence, there is a need for oral cells and tissues to efficiently detoxify xenobiotic toxicity, neutralize oxidative stress, kill invading pathogens, and eliminate inflammatory responses. In light of its potentially important biological and pharmacological activities, NAC has been advocated as a therapeutic agent in oral health care [12]. The present review focuses on the biological activities of NAC and its potential oral applications. The review also explores the potential side effects of NAC and its medical formulations. Understanding the actions of NAC and its biological effects on oral pathological processes is helpful in the design of future clinical trials and expedites clinical translation of the use of this drug in oral medicine.

## 2. Antioxidation Activity

Intracellular oxidative stress occurs when reactive oxygen species (ROS)/reactive nitrogen species (RNS) are produced beyond the cell's antioxidation capacity. Excessive oxidative stress results in oxidative modification of proteins, lipids, DNA, and subsequent cell death [13]. This process contributes to numerous pathological conditions including oral diseases [14]. Antioxidants, either natural or synthetic, are effective in diminishing the cumulative effects of oxidative stress and NAC is of particular interest. N-Acetylcysteine is a direct antioxidant that interacts with the electrophilic groups of free radicals through its free thiol side-chain. The rate constants of the reactions of NAC with various substrates under experimental conditions are summarized in Table 1. Because NAC reacts rapidly with hydroxyl radical (^·^OH), nitrogen dioxide (^·^NO_2_), and carbon trioxide ion (CO_3_^·−^), it detoxifies ROS produced by leukocytes [15]. Although NAC does not react directly with nitric oxide (NO), it reacts with its reduced and protonated form, nitroxyl (HNO) [16]. In addition, NAC chelates transition metal ions such as Cu^2+^ and Fe^3+^, as well as heavy metal ions such as Cd^2+^, Hg^2+^, and Pb^2+^, through its thiol side-chain to produce complexes. This chelation process facilitates removal of these metal ions from the body [17].

Apart from its role as a direct antioxidant, NAC also functions as an indirect antioxidant. The rate constants of the reactions of NAC with superoxide (O_2_^·−^), hydrogen peroxide (H_2_O_2_), and peroxynitrite (ONOO^−^) are relatively low under physiological conditions. The indirect antioxidation action of NAC relies on replenishment of intracellular GSH, the body's major antioxidant with versatile cellular functions (see [18] for review). Considering the overwhelming antioxidation potential of GSH and the very low concentrations of NAC inside cells, it is likely that the predominant antioxidation effects of NAC are associated with maintaining GSH levels in the intracellular environment [19].

**Figure 3:**
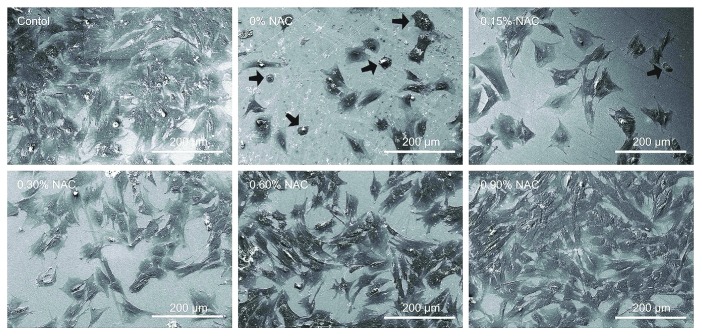
Representative scanning electron microscopy images showing attachment and morphology of human dental pulp cells on the surface of poly(methyl methacrylate) resin in the presence or absence of N-acetylcysteine (NAC). After culturing for 24 hours, human dental pulp cells grew poorly with round or collapsed appearances in subgroup 0 wt.% NAC and subgroup 0.15 wt.% NAC (arrows). In contrast, the cells attached and spread well with spindle or polygonal shapes in subgroups 0.3 wt.%, 0.6 wt.%, and 0.9 wt.% NAC. The number of adhering cells increased as the concentration of NAC increased in the experimental poly(methyl methacrylate) resin. Similar to the control, the resin surface of subgroup 0.9 wt.% NAC was almost fully covered by cells. Reprinted with permission [31].

A plethora of *in vitro* and *in vivo* studies have demonstrated the protective effectiveness of NAC against various oxidative insults in the oral cavity. These insults include blue light irradiation [20], exposure to fluoride [21], H_2_O_2_ [22] and NO [23], and lipopolysaccharides [24], as well as dental and implantable materials [25] (Table 2). Residual monomers released from resin restorations due to incomplete polymerization could cause adverse biological reactions in oral tissues [26]. Based on *in vitro* studies of multiple target cells, resin monomers were detected to induce cytotoxic and genotoxic effects and specifically interfere with various vital cellular functions [27]. Although the exact mechanism is still largely unknown, many prior reports suggest that these adverse effects are associated with monomer-induced oxidative stress as a consequence of the formation of ROS and concomitant with depletion of GSH [6]. Based on the findings that disturbance of intracellular redox balance is involved in the cytotoxic effects of resin monomers, NAC has been used and identified as an effective molecule to reduce such cytotoxicity [28]. At first, it was believed that NAC exerts protective effects against monomer-related cytotoxicity mainly through its antioxidative properties by directly scavenging over-produced ROS, meanwhile replenishing the exhausted intracellular GSH. However, very recently, some researchers have suggested a further relevant protective mechanism by providing evidence showing that NAC can directly react with the methacrylic group of resin monomers through Michael-type addition reaction thus reducing the availability of free dental resin monomers [29, 30]. Accordingly, NAC has been incorporated into poly(methyl methacrylate) (PMMA) dental resin. Addition of 0.15 weight percent (wt.%) NAC remarkably improves the biocompatibility of PMMA resin without exerting significant adverse influence on its mechanical properties [31] (Figure 3). NAC has also been shown to enhance differentiation of osteoblastic cells *in vitro* and accelerate bone healing when added to a collagenous sponge implanted in rat femoral critical size defects [32, 33]. These data highlight the potential of NAC for clinical application as an osteogenic enhancer in bone regeneration therapies. Significantly higher salivary ROS, lipid peroxidation, and NO and nitrite levels are present in oral lichen planus patients [34], suggesting antioxidants such as NAC have therapeutic potential in managing this disease.

## 3. Anti-Inflammatory Activity

**Figure 4:**
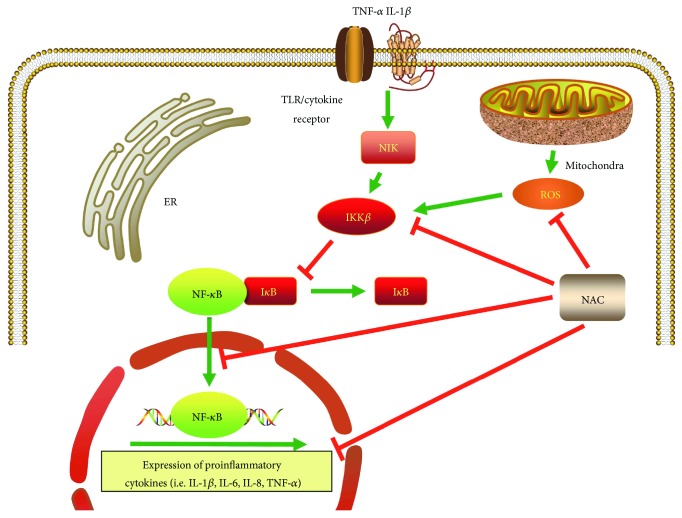
Model of the anti-inflammatory activity of N-acetylcysteine. NF-*κ*B is naturally bound to I*κ*B that prevents its nuclear translocation. Phosphorylation of I*κ*B by IKK*β* results in dissociation of I*κ*B from NF-*κ*B. This process facilitates nuclear translocation of NF-*κ*B as well as transcription of genes involved in the inflammation cascade and immune response. N-Acetylcysteine prevents activation of NF-*κ*B by removal of ROS, inhibition of IKK*β*, and nuclear translocation of NF-*κ*B. N-Acetylcysteine also inhibited the synthesis of proinflammatory cytokines such as IL-1*β*, IL-6, IL-8, and TNF*α*. ER: endoplasmic reticulum; I*κ*B: inhibitor of NF-*κ*B; IKK*β*: inhibitor of *κ*B kinase; IL: interleukin; NF-*κ*B: nuclear factor kappa-light-chain-enhancer of activated B cells; NIK: NF-*κ*B-inducing kinase; ROS: reactive oxygen species; TLR: toll-like receptor; TNF-*α*: tumor necrosis factor-*α*.

Another potential therapeutic application of NAC stems from its anti-inflammatory activity (Figure 4). The transcription factor NF-*κ*B plays a critical role in many aspects of the inflammation cascade and immune response by regulating the expression of related genes [35]. The anti-inflammatory effect of NAC is associated with the decrease of NF-*κ*B activity; NAC suppresses ubiquitination and degradation of I-*κ*B (an inhibitor of NF-*κ*B) and thereby blocks NF-*κ*B nuclear translocation and activation [36, 37]. As a direct antioxidant and GSH precursor, NAC scavenges free radicals and inhibits upstream NF-*κ*B-activating events [38]. N-Acetylcysteine also modulates transcription activities through several pathways involving c-Fos/c-Jun, STAT, and cyclin inhibitors [39]. In oral inflammation, NAC prevents expression of lipopolysaccharide-induced proinflammatory cytokines such as interleukin-1*β* (IL-1*β*), IL-6 and IL-8, tumor necrosis factor-alpha (TNF-*α*), and transforming growth factor *β* (TGF- *β*) in macrophages [40] and gingival fibroblasts [41]. Restorative resin materials may cause inflammatory responses by monocyte activation and changes in the levels of released cytokines. This is demonstrated by augmented proinflammatory cytokine levels in the gingival crevicular fluid [42]. N-Acetylcysteine has been used to prevent inflammation in cytotoxicity studies of resinous materials [43]. Oral administration of NAC decreases alveolar bone loss in a dose-dependent manner in a rat model of experimental periodontitis [44]. Considering that NAC acts as an osteogenesis-enhancing molecule [12], NAC-loaded nanotube titanium dental implants have been developed that are capable of enhancing bone regeneration and osseointegration through sustained release of NAC [45]. The loaded NAC increased the hydrophilicity of the implant surface, thereby facilitating osteoblast adhesion and proliferation. The NAC released from the loaded nanotubes also inhibits lipopolysaccharide-induced oxidative stress and inflammatory cytokines, as well as reduces expression of receptor activator of nuclear factor kappa B ligand (RANKL). These findings support the use of NAC-loaded nanotube titanium dental implants in clinical applications, although their immunomodulatory activities require further substantiation. Nevertheless, it has been reported that long-term, low-dose NAC application increases the expression of proinflammatory cytokines in lipopolysaccharide-stimulated macrophages through enhancement of kinase phosphorylation [46].

## 4. Antimicrobial Activity

Although NAC is not an antibiotic, it possesses antimicrobial properties. Since the initial demonstration of inactivation of *Staphylococcus epidermidis* biofilm formation by NAC in 1997 [47], many studies have demonstrated the efficacy of NAC in reducing biofilm formation induced by a broad array of medically important microorganisms (Table 3). One of those studies evaluated the antibacterial and biofilm eradication potential of NAC on *Enterococcus faecalis* [48], one of the most important opportunistic pathogens responsible for persistent root canal infections [49]. In that study, the authors demonstrated that NAC was effective against both the planktonic and biofilm forms of *E. faecalis*; antimicrobial efficacy was not reduced by the presence of dentin powder for up to 14 days. A more recent study reported that NAC has potent antibacterial effects against planktonic endodontic pathogens (*Actinomyces naeslundii*, *Lactobacillus salivarius*, *Streptococcus mutans*, and *E. faecalis*) and effectively inhibits biofilm formation by all the monospecies and multispecies bacteria [50]. Eradication of mature multispecies biofilms was also observed by scanning electron microscopy after a 10 min treatment with NAC at concentrations of 25 mg/mL or higher. The biofilm disrupting activity of NAC is significantly higher than that of saturated calcium hydroxide or 2% chlorhexidine.

During root canal treatment, it is essential to eradicate residual bacterial infections from the root canal system with intracanal medicaments such as chlorhexidine or calcium hydroxide. Although chlorhexidine exhibits substantivity, it is inactivated by dentin and has a limited ability to penetrate the deep layer of biofilms [51]. Calcium hydroxide, on the other hand, decreases the bond strength of resin-based endodontic sealer to dentin [52] and is less effective against *E. faecalis* and *Candida albicans* [53]. Because NAC possesses anti-inflammatory effect on lipopolysaccharide-induced inflammatory responses [40] and analgesic property for relieving postendodontic pain that is comparable to the effect of ibuprofen [54], it has immense potential to be used as an alternate intracanal medicament in root canal treatment. Some research groups have combined additional components with NAC to achieve augmented or broad-spectrum antimicrobial applications. These additional components include alexidine [55], chlorhexidine [56], taurolidine [57], and other antibiotics [58]. Despite the potent antimicrobial efficacy of NAC, when used alone or in association with antibiotics in oral cavity infections, few studies to date have evaluated the antimicrobial activity of NAC using animal models. In a murine experimental periodontitis model, a dose-dependent reduction was observed in the invasion of *Fusobacterium nucleatum* in immortalized human gingival epithelial cells by NAC [59]. This is achieved by inhibition of *F. nucleatum*-induced activation of Rac1, an important regulator of actin cytoskeleton dynamics responsible for the bacterial invasion of host cells [60]. Furthermore, NAC completely eliminates experimental periodontitis induced in mice by the periodontal pathogens *Prevotella gingivalis* and *Treponema denticola* [59]. Although extensive efforts have been made in this field, the exact mechanisms responsible for the antimicrobial and antibiofilm activities of NAC are still speculative. These speculations include (1) inhibition of cysteine utilization in bacteria, (2) reaction between the thiol group of NAC and bacterial cell proteins, (3) reduction of bacterial extracellular polymeric substances that are responsible for bacterial adhesion and pathogenicity, and (4) disturbance of intracellular redox equilibrium with potential indirect effects on cell metabolism and intracellular signal transduction pathways [61, 62].

NAC also shows its therapeutic potential for wound healing and tissue regeneration. It was shown that NAC exerted the bacteriostatic effects on wound pathogens such as *Staphylococcus aureus* and *Streptococcus pyogenes* both in brain heart infusion (BHI) broth and on agar *in vitro* [63]. Addition of NAC to the collagen scaffold was shown to protect gingival fibroblasts and bone marrow-derived osteoblasts from bacterial infection by coincubation with *S. aureus* or *S. pyogenes* and preserve bacteria-induced impairment of fibroblastic viability, attachment, adhesion behavior, and osteoblastic differentiation. In addition, NAC assists the cells' ability to diminish the damaging effects of ROS and reduce inflammation during wound healing [64]. NAC was beneficial for treating grave burn injuries in a rat comb burn model when administered via the oral or intraperitoneal route [65]. The effects on wound healing of nasal mucosa were also confirmed, when NAC was intraperitoneally administered to rats with nasal trauma [66]. Experimental rat skin wounds were effectively treated with topical NAC, and the efficacy of NAC in wound healing was comparable to dexpanthenol, a molecule widely used to improve wound healing [67]. NAC has also been functionalized as a scaffold with anti-infective capabilities, thus assisting healing of soft and hard tissues. Recently, a topically administered eye drop (Lacrimera®) based on chitosan-N-acetylcysteine (C-NAC) has been recently introduced and received CE marking in Europe. This eye drop has been shown to effectively improve corneal wound healing in a rabbit model of corneal epithelial debridement [68].

## 5. Anticarcinogenic Activity

Since the first report on the anticarcinogenic function of NAC in 1984 [69], modulation of genotoxicity, oncogenicity, and tumor progression processes by NAC has been extensively studied in cellular experiments, animal models, and human clinical trials by independent researchers. It has become apparent that NAC exerts its anticarcinogenic actions by a broad array of mechanisms including the attenuation of genotoxic ROS, modulation of metabolism and mitochondrial pathways, induction of DNA repair, inhibition of genotoxicity and cell transformation, modulation of signal transduction pathways, regulation of cell survival and apoptosis, anti-inflammatory activity, immunological effects, influence on cell cycle progression, antiangiogenetic activity, and inhibition of invasion and metastasis [70].

Oral cancer is one of the most frequently diagnosed cancers worldwide. This type of cancer constitutes 90% of head and neck cancers and involves squamous cell carcinomas of several anatomical sites such as the lip and oral cavity, pharynx, and larynx. According to the American Cancer Society, approximately 30,000 new cases of oral cancer are diagnosed in the United States alone in 2015, of which 5990 cases are fatal [71]. Despite technical advances in treatment modalities such as surgery, radiotherapy, and chemotherapy, the prognosis of oral cancer remains inauspicious; the estimated 5-year overall survival is only 56% [72]. Major risk factors associated with the development of oral cancer include smoking, tobacco chewing, alcohol consumption, and betel nut chewing. Focusing on the potential positive effects of NAC on smoke-related carcinogenesis, a phase II trial (EUROSCAN) was conducted on 2592 patients suffering from head and neck cancer or lung cancer, most of whom were former or current smokers. No statistically significant improvement in terms of survival, event-free survival, or tumor remission was observed in those patients after a 2-year supplement of NAC (600 mg/day) [73]. By contrast, several studies reported the ability of NAC to exert protective effects against preneoplastic lesions, benign tumors, and/or malignant tumors in animal tumorigenesis models induced by individual cigarette smoke components [74]. A randomized double-blind phase II chemoprevention trial was conducted on 41 healthy smoking volunteers. After 6 months of oral NAC (2 × 600 mg/day), significant decrease in the investigated biomarkers was observed, including the levels of bulky DNA adducts and 8-hydroxy-2′-deoxyguanosine in bronchoalveolar lavage cells, as well as the frequency of micronuclei in mouth floor and soft palate cells [75]. The unfavorable clinical outcome of oral cancer is often associated with aberrant activation of epidermal growth factor receptor (EGFR) signaling [76]. Encouraged by the observation that NAC suppressed EGFR-induced phosphorylation in an earlier study [77], the effects of NAC in EGFR-overexpressing invasive oral cancer was conducted on cancer cell growth in a murine xenograft model [78]. The authors found that NAC suppresses growth of cancer cells by mediating the EGFR/Akt/HMG box-containing protein 1 signaling pathway in oral cancer cells, as well as tumor growth. N-Acetylcysteine has also been investigated as a potential agent to attenuate the side effects of platinum-based chemotherapy. By suppressing oxidative stress and oxidation-associated signals, NAC was found to reduce cisplatin-induced acute renal failure in rats [79]. A pilot randomized study with 13 head and neck cancer patients reported that transtympanic injections with NAC prior to cisplatin exposure appears to prevent cisplatin-induced ototoxicity, although better delivery is required to improve the efficacy of this treatment modality [80]. The protective effect of NAC is believed to be achieved by binding directly to cisplatin molecules and acting as free radical scavengers.

## 6. Potential Side Effects of NAC and Its Formulations

Although NAC-based therapeutics has been advocated for oral health care, proactive approximations are required to establish safety conditions and appropriate delivery formulations. N-Acetylcysteine has a long-established safety record in adults and children; the drug has been approved by the US Food and Drug Administration since 1963. The adverse effects experienced with the use of NAC are somewhat dependent on the route of administration. The pharmacokinetics and pharmacodynamics of NAC have been investigated in a phase I clinical study of 26 volunteers with a 6-month oral administration of NAC. The major reported side effects were gastrointestinal symptoms including intestinal gas, diarrhea, nausea, and fatigue with the highest nontoxic dose being 800 mg/m^2^/day [81]. In another clinical trial, oral administration of NAC at doses up to 8000 mg/day was reported to cause no significant adverse reactions in patients infected with the human immunodeficiency virus [82]. In contrast, severe anaphylactoid symptoms such as flushing, pruritus, angioedema, bronchospasm, and hypotension have been reported after intravenous administration of NAC. These symptoms are likely to be attributed to the transient high plasma concentrations of NAC and are most prevalent immediately after the initial loading infusion; the symptoms subside rapidly after administration is discontinued [1]. Nevertheless, severe systemic reactions are uncommon. Considering the poor oral absorption of dietary GSH, orally administered NAC has been found to be more efficient than direct GSH administration and is as effective as intravenously administered NAC [83]. Compared with cysteine, the acetyl moiety of NAC reduces the reactivity of the thiol functionality, rendering NAC less toxic and less susceptible to oxidation to disulfide and easier for absorption and distribution [84]. N-Acetylcysteine is rapidly and almost completely absorbed after oral administration in both animals and humans; only 3% of radioactive-labeled NAC is excreted in the feces [85]. Thus, NAC is a better source of cysteine compared with parenteral administration of cysteine. Several *in vitro* studies demonstrated that extremely high NAC concentrations (typically 10 mM and sometimes as high as 100 mM) alter protein structure and function, such as modulation of angiotensin II receptor binding [86] and TNF-*α* blocking by reducing the affinity of its receptor [87]. Collectively, the toxicity associated with NAC therapy does not appear to be a negligible issue. Oral administration is preferred despite some clinical situations where other drug delivery routes are required. A number of orally administered NAC formulations are commercially available, including Mucomyst™ (Brisol-Myers Squibb Co., Princeton, NJ, USA) as an antidote for acetaminophen overdose, PharmaNAC® (BioAdvantex Pharma Inc., Mississauga, ON, Canada), and several formulations packaged in pill and tablet forms in Europe. Several companies also manufacture and sell NAC in combination with other daily nutritional supplements such as multivitamins and antioxidants (e.g., Swanson Health Products, Fargo, ND, USA). It is important to note that the manufacture of NAC requires prevention of NAC oxidation to the disulfide dimer *N,N*′-diacetylcystine. Unlike NAC, the latter is pharmacologically active and causes immunologic effects at very low concentrations [88]. According to the European Good Manufacturing Practice standards, *N,N*′-diacetylcystine should constitute less than 0.1% of commercialized NAC formulations [89].

## 7. Conclusions and Prospects

The past decade has witnessed an explosion of data regarding the multifaceted biological activities of NAC, including antioxidant, anti-inflammatory, antimicrobial, and anticarcinogenic activities. The oral cavity has continuously challenged various environmental insults that are likely to generate oxidative stress, induce inflammation, and even initiate cancer. The biological and pharmacological activities of NAC and its ability to circumvent the mechanisms of disease progression make it a potential therapeutic agent for intervention in dental and oral disorders. Still, its clinical effectiveness needs further investigations, since most of the results in this area of research are derived from *in vitro* and *in vivo* studies. The focus of future research should be the following: (i) to develop novel dental and implantable materials with improved biocompatibility by incorporating NAC, (ii) to investigate whether NAC could be used alone or with other drugs to treat oral lichen planus, (iii) to examine NAC clinically to be used as an alternate intracanal medicament in root canal treatment, (iv) to examine the clinical effectiveness of NAC for the treatment of wound healing, and (v) to evaluate the clinical application of NAC as an anticancer adjuvant for oral cancer treatment.

## Figures and Tables

**Table 1:** Rate constants of N-acetylcysteine reactions with representative compounds (adapted from [12]).

Compounds	Rate constant (M^−1^ s^−1^)	Experimental conditions	Reference
CO_3_^·−^	≈1.0 × 10^7^ 1.8 × 10^8^	pH = 7; RT pH = 12; RT	[90]
HNO	5.0 × 10^5^	pH = 7.4; 37°C	[91]
HOCl	>10^7^	pH ≈ 7.4; 21–24°C	[92]
HOSCN	7.7 × 10^3^	pH = 7.4; 22°C	[93]
H_2_O_2_	0.16 ± 0.01 0.85 ± 0.09	pH = 7.4; 25°C pH = 7.4; 37°C	[94] [95]
^·^NO_2_	≈2.4 × 10^8^ ≈1.0 × 10^7^	pH > pK_a_; RT pH = 7.4; RT	[96]
O_2_^·−^	68 ± 6 <10^3^	pH = 7; RT pH = 7.4; 25°C	[97] [95]
^·^OH	1.36 × 10^10^	pH = 7; RT	[95]
ONOO^−^	415 ± 10	pH = 7.4; 37°C	[98]

RT: room temperature.

**Table 2:** Representative studies on the protective effects of N-acetylcysteine against various oxidative insults in the oral cavity.

Insult	Cell model	Mode of action	NAC dose	NAC function	Reference
Co-Cr dental alloys	Human gingival fibroblasts, human osteoblasts	ROS ↑, TNF-*α* ↑, IL-1*β* ↑, IL-6 ↑, IL-8 ↑, iNOS ↑, NO ↑, COX-2 ↑, PGE2 ↑, Nrf2 ↑, NQO ↑, HO-1 ↑, GST ↑, GR ↑, GCL ↑, p-JAK2 ↑, p-STAT3 ↑, p-p38 MAPK ↑, p-ERK ↑, p-JNK ↑, NF-*κ*B p65 ↑	20 mM	NAC pretreatment inhibited Co-Cr alloy-induced proinflammatory cytokine production and NF-*κ*B activation	[99]
Dental resin monomers(e.g., HEMA, TEGDMA, MMA)	Human dental pulp cells	ROS ↑, GSH ↓, MDA ↑, SOD ↓, CAT ↑, GPx ↓, mitochondria dysfunction, intrinsic mitochondrial apoptosis	10 mM	NAC remarkably relieved dental resin monomer-induced oxidative stress and subsequently protected the cells against apoptosis	[25]
Dental quaternary ammonium monomer(e.g., DMAE-CB)	Human dental pulp cells, mouse fibroblasts	ROS ↑, cell cycle arrest, mitochondria dysfunction, intrinsic mitochondrial apoptosis	10 mM	NAC could reduce the cytotoxicity of quaternary ammonium monomers	[29, 100]
Dentin bonding agents	Human dental pulp cells	ALP ↓, DSPP ↓, OCN ↓, matrix, mineralization ↓	5 mM	NAC was useful for reversing cytotoxicity and antidifferentiation effects of dentin bonding agents on human dental pulp cells	[101]
Mineral trioxide aggregate (MTA)	Rat dental pulp cells	ROS ↑, GSH ↓	5 mM	The addition of NAC improved the number and spreading behavior, reduced ROS production, and increased the cellular antioxidant resources of rat dental pulp cells cultured on MTA	[102]
Root canal sealers	Mouse osteoblastic cell line	GSH ↓	10 mM	NAC prevented cytotoxicity and intracellular GSH depletion of root canal sealers	[103]
Photoinitiators(e.g., CQ)	Human dental pulp cells	ROS ↑, collagen I ↓, p21 ↑, HO-1 ↑, COX-2 ↑, p-ATM ↑, p-Chk2 ↑, p-p53 ↑, GADD45*α* ↑, 8-isoprostane ↑, PGE2 ↑, cell cycle arrest, apoptosis	2.5 mM,5 mM	NAC prevented CQ-induced cytotoxicity, cell cycle arrest, apoptosis and PGE_2_ production of pulp cells	[104]
Fluoride exposure	Rat hepatocytes	MDA ↑, SOD ↓, GPx ↓, GR ↑, GSH ↓, TAS ↓	1 mM	NAC pretreatment provided protection against fluoride-induced oxidative stress	[105]
Heat stress	Human dental pulp cells	ROS ↑, IL- 8↑, IL-8R ↑, HO-1 ↑, nuclear Nrf2 ↑, cytosolic Nrf2 ↓, SOD ↑, HO-1 ↑, GST ↑, GCL ↑, GR ↑	20 mM	The addition of NAC to cells blocked heat stress-activated proinflammatory chemokines and Nrf2-mediated antioxidant responses	[10]
Hydrogen peroxide (H_2_O_2_)	Rat palatal mucosal cells	Apoptosis, collagen I ↓, collagen III ↓, P4H ↓, GSH ↓, GSSG ↑	2.5 mM,5 mM,10 mM	NAC substantially reduced H_2_O_2_-induced elevation of cellular proliferation and collagen production associated with an increase in intracellular GSH reserves and decrease in GSSG	[22]
Lipopolysaccharide (LPS)	Human gingival fibroblasts	ROS ↑, GSH/GSSG ↓, IL-1*β* ↑, IL-6 ↑, IL-8 ↑, TNF-*α* ↑, MMP2 ↑	10 mM,20 mM	NAC prevented LPS-induced proinflammatory cytokines and MMP2 production	[41]
Mechanical stress	Human dental pulp cells	ROS ↑, IL-1*β* ↑, IL-6 ↑, IL-8 ↑, TNF-*α* ↑, HO-1 ↑, NQO-1 ↑, GPx ↑, SOD ↑, Nrf2 ↑	20 mM	NAC prevented the production of proinflammatory cytokines and ROS, as well as the activation of subsequent Nrf2-mediated gene transcription in response to mechanical strain	[11]
Nitric oxide (NO)	Human dental pulp cells	ROS ↑, intrinsic mitochondrial apoptosis	5 mM	NAC rescued the cell viability decreased by NO and downregulated NO-induced activation of proapoptotic mitochondria-dependent pathways	[23]

ALP: alkaline phosphatase; ATM: ataxia-telangiectasia mutated; CAT: catalase; Chk2: checkpoint kinase 2; Co: cobalt; COX-2: cyclooxygenase-2; CQ; camphorquinone; Cr: chromium; DSPP: dentin sialophosphoprotein; DMAE-CB: methacryloxylethyl cetyl ammonium chloride; ERK: extracellular signal-regulated kinase; GADD45*α*: growth arrest and DNA damage-inducible protein GADD45 alpha; GCL: *γ*-glutamylcysteine lygase; (GPx: glutathione peroxidase; GR: glutathione reductase; GSH: reduced glutathione; GSSG: oxidized form of glutathione; GST: glutathione S-transferase; HEMA: 2-hydroxyethyl methacrylate; HO-1: heme oxygenase 1; H_2_O_2_: hydrogen peroxide; IL-1*β*: interleukin-1beta; iNOS: inducible nitric oxide synthetase; JNK: c-Jun N-terminal kinase; LDH: lactate dehydrogenase; LPS: lipopolysaccharide; MDA: malondialdehyde; MMA: methyl methacrylate; MMP: matrix metalloproteinase; MTA: mineral trioxide aggregate; NAC: N-acetylcysteine; NF-*κ*B: nuclear factor kappa-light-chain-enhancer of activated B cells; NO: nitric oxide; NQO: nitroquinoline 1-oxide; Nrf2: NF-E2-related factor 2; OCN: osteocalcin; PGE2: prostaglandin E2; p21: cyclin-dependent kinase inhibitor 1; p38 MAPK: p38 mitogen-activated protein kinase; P4H: prolyl-4 hydroxylase; p-JAK2: phosphorylation of janus kinase 2; ROS: reactive oxygen species; SOD: superoxide dismutase; STAT3: signal transducer and activator of transcription 3; TAS: total antioxidant status; TEGDMA: triethylenglycol dimethacrylate; TNF-*α*: tumor necrosis factor-alpha.

**Table 3:** Representative studies on antimicrobial and antibiofilm activities of N-acetylcysteine against various oral pathogenic microorganisms.

Pathogens examined	NAC concentrations (mg/mL)	Related niche	Reference
Gram-positive bacteria			
*Actinomyces naeslundii*	1.56–25	C/E	[50]
*Enterococcus faecalis*	1.56–50	E	[48]
	1.56–25	E	[50]
	2.5–20	E	[57]
*Lactobacillus salivarius*	1.56–25	C	[50]
*Staphylococcus aureus*	20	C	[106]
	6–24	C	[107]
	80	C	[108]
	2–4	C	[109]
	80	C	[110]
*Staphylococcus epidermidis*	4–40	C	[111]
	0.03–2	C	[112]
	4–40	C	[113]
	80	C	[108]
	2–4	C	[109]
	0.5–32	C	[114]
	80	C	[110]
	0.003–8	C	[47]
*Streptococcus mutans*	0.78–6.25	C/E	[50]
Gram-negative bacteria			
*Acinetobacter baumannii*	0.25–2	C/E	[62]
*Enterobacter cloacae*	80	E	[108]
	0.25–2	E	[62]
*Escherichia coli*	2–4	C/E/P	[109]
	0.007-8	C/E/P	[115]
*Klebsiella pneumoniae*		E	[106]
		E	[108]
		E	[109]
		E	[110]
		E	[62]
*Prevotella intermedia*	0.375–3	E/P	[58]
*Proteus* spp.	2.5	C/E/P	[106]
	2–4	C/E/P	[109]
*Pseudomonas aeruginosa*	2.5	C/E/P	[106]
	12.5	C/E/P	[116]
	3–24	C/E/P	[107]
	80	C/E/P	[108]
	0.5–10	C/E/P	[117]
Yeasts			
*Candida albicans*	0.5–32	C/E/P	[114]
	0.312–40	C/E/P	[118]

C: caries; E: endodontic infections; P: periodontitis.
